# Microglia, seen from the CX_3_CR1 angle

**DOI:** 10.3389/fncel.2013.00026

**Published:** 2013-03-18

**Authors:** Yochai Wolf, Simon Yona, Ki-Wook Kim, Steffen Jung

**Affiliations:** Department of Immunology, The Weizmann Institute of ScienceRehovot, Israel

**Keywords:** microglia, neuropathology, Cre-loxP knock-in mice, CX_3_CR1, neuroimmunology

## Abstract

Microglial cells in brain and spinal cord are characterized by high expression of the chemokine receptor CX_3_CR1. Expression of the sole CX_3_CR1 ligand, the membrane-tethered and sheddable chemokine CX_3_CL1/fractalkine, is restricted in the brain parenchyma to selected neurons. Here we summarize our current understanding of the physiological role of CX_3_CR1 for microglia function and the CX_3_C axis in microglial/neuronal crosstalk in homeostasis and under challenge. Moreover, we will discuss the efforts of our laboratory and others to exploit CX_3_CR1 promoter activity for the visualization and genetic manipulation of microglia to probe their functional contributions in the central nerve system (CNS) context.

## Introduction

Microglia are members of the mononuclear phagocyte system alongside other macrophages, monocytes and dendritic cells (Geissmann et al., 2010). Sequestered behind the blood brain barrier (BBB) in the unique neuronal/macroglial context, microglia display a gene expression profile that significantly differs from other tissue macrophages (Gautier et al., 2012). Moreover, highlighting its independence the microglia compartment is established well before birth from a primitive hematopoietic wave and subsequently maintains itself throughout adulthood through longevity and limited self-renewal (Alliot et al., 1999; Ginhoux et al., 2010). Microglia share this prenatal establishment with other tissue macrophage populations; however the latter seem less secluded and more promiscuous with respect to the incorporation of monocytic cells derived from the fetal liver or during challenge (Hoeffel et al., 2012; Yona et al., 2013). In contrast, even after a prominent trauma-associated influx of monocytes into the injured central nerve system (CNS), these cells do not seem to permanently seed the brain. Rather, the steady state relying solely on microglia seems to be restored (Ajami et al., 2011). Steady state microglia are distributed throughout the CNS, including brain and spinal cord, although there is evidence for considerable region specific differences in density, phenotype and responsiveness (de Haas et al., 2008). As immune cells, microglia are sensors of injury and pathologic conditions (Hanisch and Kettenmann, 2007). More recent data have furthermore revealed critical microglia contributions during CNS development and brain homeostasis (Tremblay et al., 2011). Much of our knowledge about microglia biology currently relies on data obtained from *in vitro* cultured cells. Under these conditions microglia might however loose much of their uniqueness and turn into prototype macrophages. This calls for the development of experimental systems that will allow the study of microglia in their unique physiological CNS environment.

**Figure 1:**
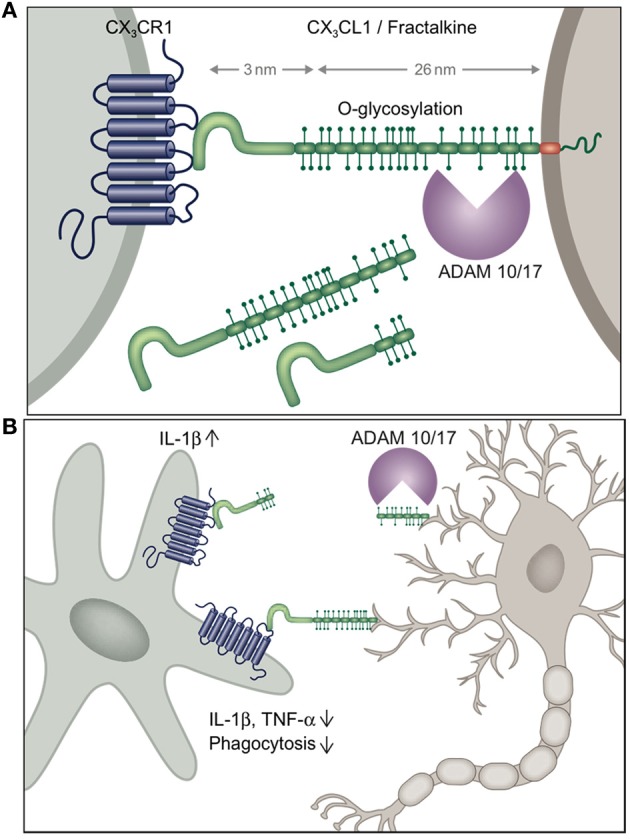
**(A)** Schematic of CX_3_C chemokine family and **(B)** potential scenario of differential outcomes of neuronal shed and membrane-anchored CX_3_CL1 engagement by microglia inducing or suppressing microglial IL-1β production, respectively.

**Figure 2:**
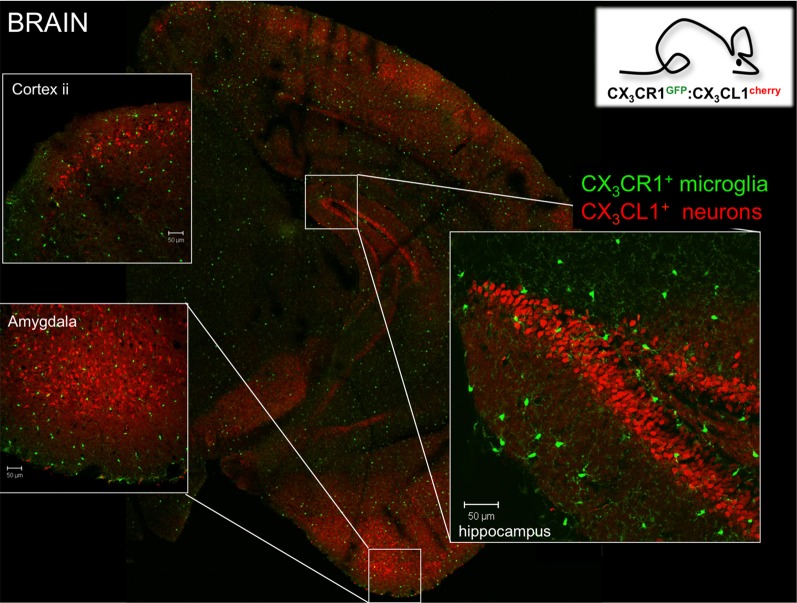
Brain section of CX_3_CR1^gfp^:CX_3_CL1^cherry^ double reporter animals (Kim et al., 2011) highlighting CX_3_CR1-expressing microglia and subsets of CX_3_CL1-expressing neurons in specific brain regions. Note that both reporters are expressed in the cytoplasm and not as fusion proteins. Thus they reflect the respective promoter activity but not the presence of the respective proteins. Hence no co-localization has to be expected.

Microglia are characterized by prominent expression of the chemokine receptor CX_3_CR1. According to the current chemokine nomenclature, which is based on the spacing of N-terminal cysteines, the chemokine CX_3_CL1/fractalkine and its sole receptor CX_3_CR1 constitute their own CX_3_C “family” (Bazan et al., 1997; Imai et al., 1997; Pan et al., 1997). CX_3_CR1 is a conventional Gα_i_-coupled seven-transmembrane receptor. Its ligand CX_3_CL1 differs however from conventional small peptide chemokines by the fact that it is synthesized as a trans-membrane protein with the CX_3_C chemokine domain displayed on an extended highly glycosylated, mucin-like stalk (Bazan et al., 1997; Pan et al., 1997) (Figure 1A). To date, CX_3_CL1 shares this unique membrane anchorage only with one other chemokine, the CXCR6 ligand CXCL16 (Matloubian et al., 2000). Proteolytic cleavage of CX_3_CL1 by the disintegrin-like metalloproteinase ADAM10 results in constitutive release of different sized shed CX_3_CL1 entities (Hundhausen et al., 2003). Moreover, under inflammatory conditions, CX_3_CL1 shedding is also promoted by ADAM17/TACE (Garton et al., 2001; Tsou et al., 2001). Aside from the prominent expression in the mononuclear myeloid compartment (Jung et al., 2000), CX_3_CR1 receptor expression has also been reported for an NK cell subset and certain T cell populations (Imai et al., 1997). Expression of the ligand CX_3_CL1 outside the CNS has been reported for intestinal epithelium and endothelium, potentially restricted to inflammatory settings (Muehlhoefer et al., 2000; Kim et al., 2011). Although CX_3_CL1 and CX_3_CR1 are hence widely distributed throughout the organism, their expression in given tissues is often highly cell type-specific. This is particularly evident in the CNS, where CX_3_CR1 expression is restricted to microglia and CX_3_CL1 expression is confine to particular neurons (Nishiyori et al., 1998; Hughes et al., 2002; Tarozzo et al., 2003). This is best highlighted in CNS sections of double reporter animals, that combine a CX_3_CR1^gfp^ locus (Jung et al., 2000) with a BAC transgene harboring a CX_3_CL1-promoter driven gene encoding a red fluorescent cherry reporter (Figure 2) (Kim et al., 2011). In CX_3_CL1^cherry^/CX_3_CR1^gfp^ mice, mCherry^+^ neurons are NeuN^+^ DCX^−^ mature neurons which are located in spatially specific regions of the brain (Figure 2), with restricted expression in the hippocampus, striatum and cortical layer II of the cerebral cortex, as well as dorsal horn neurons in the spinal cord (Kim et al., 2011). The exact nature and function of these CX_3_CL1-expressing neurons remains to be deciphered.

## Seeing is believing—*in situ* visualization of microglia using CX_3_CR1^gfp^ mice

**Figure 3:**
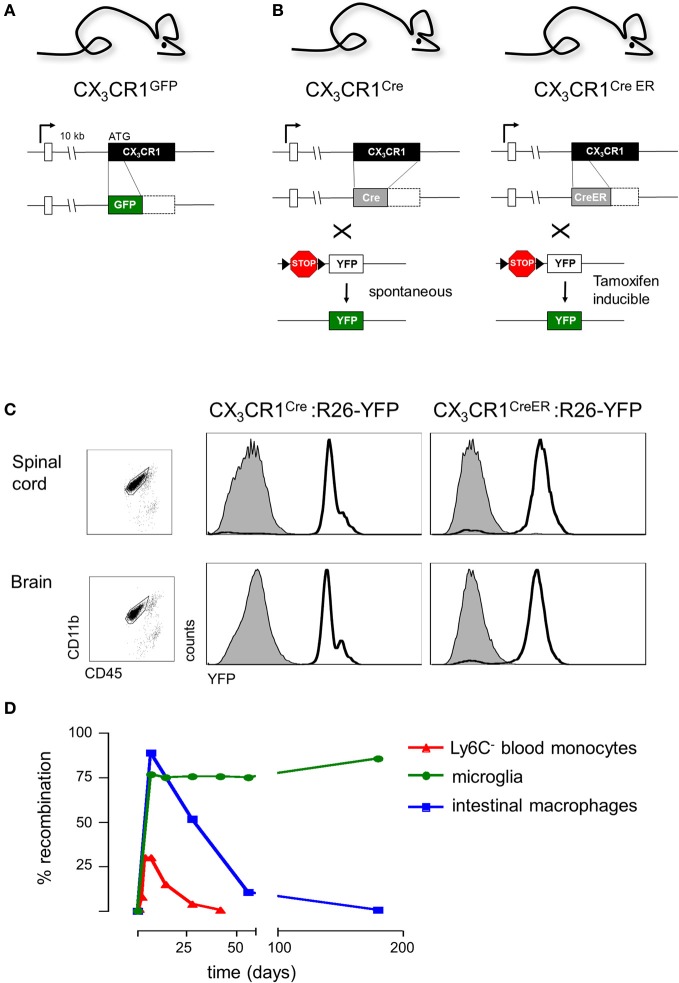
**(A)** Schematic of modified CX_3_CR1 loci of CX_3_CR1^gfp^ mice (Jung et al., 2000) and **(B)** CX_3_CR1^Cre^ and CX_3_CR1^CreER^ mice (Yona et al., 2013); **(C)** Efficient YFP labeling of both spinal cord and brain microglia in CX_3_CR1^CreER^ and CX_3_CR1^Cre^ mice crossed with R26-YFP reporter mice. CX_3_CR1^CreER^:R26-YFP mice were treated with tamoxifen to induce the rearrangement (Yona et al., 2013); **(D)** Induction but subsequent progressive loss of cells harboring gene rearrangements in peripheral myeloid cells (monocytes, intestinal macrophages) and persistence of genomic modification in microglial cells.

Microglial cells were originally identified in 1932 by the Cajal disciple del Rio-Hortega using silver staining and light microscopy. However, unraveling the role of microglia in CNS had to wait for the era of live brain imaging. Two seminal intravital microscopy studies revealed that these presumably static ramified cells are indeed highly dynamic and continuously extend fine highly motile processes that allow them to survey their immediate surrounding (Davalos et al., 2005; Nimmerjahn et al., 2005). Moreover, in response to laser-mediated lesions, microglial cells were found to rapidly respond to ATP released by astrocytes and redirected these processes toward the site of injury (Davalos et al., 2005). These pioneering imaging studies took advantage of CX_3_CR1^gfp^ mice (Jung et al., 2000) that harbor a targeted replacement of the CX_3_CR1 gene with a cDNA encoding enhanced fluorescent protein and ever since have become a popular research tool of the microglia research community. Specifically, CX_3_CR1^gfp^ mice have allowed researchers to interrogate the microglia/neuron interface at unprecedented resolution complementing electron microscopy studies with critical dynamic data (Tremblay et al., 2010). These studies revealed that microglia are in intimate contact with neuronal synapses and that these interactions are affected by visual experience (Tremblay et al., 2010). Moreover, since the insertion of the reporter gene in CX_3_CR1^gfp^ mice generates a CX_3_CR1 null locus (Jung et al., 2000) (Figure 3A), comparison of CX_3_CR1^gfp/+^ and CX_3_CR1^gfp/gfp^ mice readily allows the probing for phenotypes resulting from CX_3_CR1-deficiency using imaging strategies (see below). Of note, a potential confounding problem is the fact that when using CX_3_CR1^gfp^ mice for microglia studies, animals are heterozygotes for the chemokine receptor and microglia display due to the haplo-insufficiency considerable less CX_3_CR1 surface expression (Jung et al., 2000). However, no microglial phenotype has so far been reported for heterozygote mutant CX_3_CR1^gfp/+^ animals, when compared to mice harboring GFP transgene under the macrophage-specific ionized calcium-binding adaptor molecule 1 (Iba1) promoter (Hirasawa et al., 2005).

## Microglia and the CX_3_C axis

Analysis of receptor and ligand knock-out mice (Jung et al., 2000; Cook et al., 2001; Combadiere et al., 2003) has revealed a number of phenotypes resulting from the lack of CX_3_CR1/L1 interactions outside the CNS, including effects on artherogenesis (Liu and Jiang, 2011) and the ability of intestinal macrophages to sense gut lumen content (Niess et al., 2005). The physiological role of the CX_3_C axis and in particular mechanistic aspects, such as potential differential functions of the membrane-tethered and shed CX_3_CL1 entities, remain incompletely understood.

Of note, the membrane-tethered CX_3_CL1 molecule has a short cytoplasmic tail lacking signaling motives. Moreover, CX_3_CL1 is not known to associate with signaling competent co-receptors. Hence, it remains unclear whether interactions of microglial CX_3_CR1 and CX_3_CL1 expressed on the neuronal surface trigger a direct response in neurons. Rather, functional outcomes of CX_3_CL1 ligation with CX_3_CR1 seem to be restricted to the microglial partner, and CX_3_CR1/L1 deficiencies seem to impinge indirectly on neurons.

Below we will discuss the impacts of CX_3_CR1 and CX_3_CL1 deficiencies on microglia functions in CNS development, the maintenance of CNS homeostasis and for the robustness of the organism to handle pathological challenges.

## The CX_3_C axis in CNS development and homeostasis

Macrophages are long known to critically contribute to development and the maintenance of homeostasis (Stefater et al., 2011). The housekeeping functions of microglia are however only beginning to be appreciated to their full extent (Tremblay et al., 2011). As efficient phagocytes, microglia have emerged as key players in postnatal synaptic pruning that trim excess connections established in the developing brain for neuronal maturation. More specifically, microglia were shown to engulf presynaptic inputs during the pruning peak of retinal ganglion cells and the engulfment was dependent upon neural activity and the microglia-specific phagocytic signaling pathway involving complement receptor 3 (CR3) (Schafer et al., 2012). Interestingly, this developmental synaptic pruning by microglia seems affected in CX_3_CR1-deficient mice, which reportedly display higher number of immature dendritic spines, correlating with transiently reduced microglia densities in their two first postnatal weeks (Paolicelli et al., 2011). In the developing barrel cortex, CX_3_CR1-deficient microglia enter the thalamocortical axon cluster to a lesser degree, accompanied by delayed developmental switch of glutamate receptor subunits—a hallmark of barrel cortex development (Hoshiko et al., 2012). Here, microglia are thought to influence the maturation of synapses in a non-phagocytic, and probably soluble factor-mediated mechanism, which is yet to be elucidated. However, it remains to be established, if late maturation is directly mediated by the CX_3_C axis, or the effect seen results from impaired entry of the cells to the barrel cortex. Other approaches, such as conditional mutagenesis of microglial CX_3_CR1 will be required to distinguish between these two scenarios.

Microglia also have been shown to shape adult hippocampal neurogenesis through apoptosis-coupled phagocytosis (Sierra et al., 2010). Again, the CX_3_CR1-deficiency seems to interfere with this process, probably as a result of deleterious microglia hyper-activation. Alongside reduced hippocampal neurogenesis, CX_3_CR1^−/−^ mice were thus reported to exhibit excessive IL-1β expression and attenuated long term potentiation (LTP) resulting in impaired cognitive functions (Bachstetter et al., 2011; Rogers et al., 2011). However, others reported that CX_3_CR1^−/−^ mice have enhanced LTP and perform better in the cognitive tests, but fail to improve these cognitive functions following exposure to enriched environment, a known stimulator of neurogenesis (Maggi et al., 2011). Interestingly, the seemingly contradicting reports share the observation of reduced neuronal precursors in neurogenic niches in the absence of CX_3_CR1, which may hint at importance of CX_3_CL1 signaling for synaptic plasticity, rather than basal synaptic activity. Further substantiating the immuno-suppressive role of the CX_3_C axis, antibody-mediated blockade of CX_3_CR1 in young adult rats induced IL-1β production and decreased survival and proliferation of neural progenitor cells (Bachstetter et al., 2011). Moreover, treatment with recombinant CX_3_CL1 was found to reverse the age-related decrease in neurogenesis (Bachstetter et al., 2011).

## The CX_3_C axis and pathological settings

The CX_3_CR1 deficiency also effects neuro-inflammatory and neurodegenerative diseases, as established in murine models of Alzheimer's and Parkinson's disease (AD, PD) and amyothrophic lateral sclerosis (ALS), as well as neuropathological conditions, such as neuropathic pain and cerebral ischemia.

AD is characterized by the presence of extracellular amyloid-β peptide (Aβ) deposits surrounded by activated glia and dystrophic neurites. The CX_3_CR1-deficiency has been introduced into several established murine AD models, including the hTAu (Bhaskar et al., 2010), APP/PS1 (Fuhrmann et al., 2010; Lee et al., 2010), and CRND8 background (Liu et al., 2010). Due to neuronal activation of the p38 MAPK pathway, HTau transgenic CX_3_CR1^−/−^ mice exhibit hyper-phosphorylation of the tau protein, one of the AD hallmarks (Bhaskar et al., 2010). Again this is probably the result of excessive microglial IL-1β secretion (Bhaskar et al., 2010). Interestingly though, in combination with APP/PS1 transgenes the CX_3_CR1 deficiency reduced amyloid plaque aggregation (Lee et al., 2010). Moreover, also the two-photon microscopic comparison of APP:PS1:taup^301L^:Thy1-YFP:CX_3_CR1^GFP/+^ and APP:PS1:taup^301L^:Thy1-YFP: CX_3_CR1^gfp/gfp^ mice revealed that the CX_3_CR1 deficiency was beneficent in that it resulted in diminished microglia-mediated neuronal cell death, as well as attenuated microglia migration velocity (Fuhrmann et al., 2010). This suggests that microglia hyperactivation can have opposing outcomes in different models and potentially AD stages. Crossing the CX_3_CR1^−/−^ mice to CRND8 transgenic mice that harbor a gene encoding a mutant human amyloid precursor protein also resulted in reduced Aβ deposits, with the CX_3_CR1-deficient microglia phagocytosing amyloid plaques and displaying higher proliferation rates in the plaque regions (Liu et al., 2010). Interestingly, microglia treated *in vitro* with beta-amyloid down-regulate CX_3_CR1 with concomitant induction of IL-6 and TNF-α levels (Cho et al., 2011). Moreover, also in AD patients both CX_3_CL1 and CX_3_CR1 are reportedly down-modulated (Cho et al., 2011). In summary, disruption of the CX_3_C axis results in microglia activation that includes beneficial enhanced phagocytosis of amyloid plaques, but is also associated with potentially detrimental secretion of pro-inflammatory cytokines causing neurotoxicity.

In rodent models of LPS-induced neuroinflammation, PD and ALS, CX_3_CR1-deficient microglia were found to overexpress IL-1β and display neurotoxic activity (Cardona et al., 2006). Moreover, in the intrastriatal 6-hydroxydopamine (6-OHDA) rat model of PD administration CX_3_CL1 prevented dopaminergic neuron death in the substantia nigra (Pabon et al., 2011). The therapeutic potential of CX_3_CL1 regimens is further supported by the fact that exogenous CX_3_CR1 reduced ischemia-induced cerebral infarct size, neurological deficits, and caspase-3 activation (Cipriani et al., 2011). Interestingly, in this study CX_3_CL1-mediated neuroprotection required the adenosine system. However of note, another group reported that lack of CX_3_CR1 did not result microglial neurotoxicity, but rather significantly reduces ischemic damage and inflammation, alongside with reduced IL-1β and TNFα expression as well as smaller infarcts (Denes et al., 2008). The reason for these discrepancies remains unclear. Of note, the above-mentioned studies that evoke therapeutic potential of CX_3_CL1 administration (Bachstetter et al., 2011; Cipriani et al., 2011; Pabon et al., 2011) suggest that the soluble, shed CX_3_CL1 isoform suffices to trigger CX_3_CR1 signaling on microglia and suppress microglia activation. In support of this notion, adenovirus-mediated gene therapy that selectively expressed synthetic shed or non-sheddable CX_3_CL1 variants was shown to ameliorate neurotoxicity when injected directly into the substantia nigra of CX_3_CL1^−/−^ mice that had been subjected to a PD model initiated by MPTP neurotoxin challenge (Morganti et al., 2012).

Challenging the notion of a mere anti-inflammatory role of CX_3_CL1-induced CX_3_CR1 signaling, deficient CX_3_CR1 signaling was reported to promote recovery after mouse spinal cord injury (Donnelly et al., 2011). However, the effects observed in this study might not be directly related to microglia but rather linked to the concomitant impaired recruitment and activation of Ly6C^−^ blood monocytes (Donnelly et al., 2011), which depend on CX_3_CR1 signaling for survival (Landsman et al., 2009). Nevertheless, the authors reported that *in vitro* exposure of wt but not CX_3_CR1-deficient microglia induced their expression of IL-6, though not IL-1β (Donnelly et al., 2011).

Microglia residing in the dorsal horn of the spinal cord are critical contributors to nociceptive transmission following peripheral nerve/tissue injury (Milligan and Watkins, 2009). Shed neuronal CX_3_CL1 was proposed to be a critical mediator of spinal neuronal-microglial communication in chronic pain. CX_3_CL1 cleavage in the dorsal root ganglion (DRG) was reported to depend on microglial release of the lysosomal protease cathepsin S potentially in response to ATP release and P2X7 receptor (Clark et al., 2007, 2010). In keeping with the notion that CX_3_CL1 is an ADAM substrate (Garton et al., 2001; Tsou et al., 2001; Hundhausen et al., 2003), CX_3_CL1 could also be cleaved by the matrix metalloprotease MMP9 that was found upregulated in injured DRG primary sensory neurons (Kawasaki et al., 2008). Shed CX_3_CL1 was proposed to engage microglial CX_3_CR1 and trigger via p38 MAPK phosphorylation pain-causing migroglial IL-1β secretion (Zhuang et al., 2007). CX_3_CR1-deficient mice reportedly display unimpaired responses to acute thermal and mechanical noxious stimuli, but displayed deficits in inflammatory and neuropathic nociceptive responses in the partial sciatic nerve ligation model (Staniland et al., 2010). The CX_3_C axis also was reported to be involved in the IL-1β-induced hyperalgesia observed in animals that have a microglia-restricted reduction of the G protein-coupled receptor kinase 2 (GRK2) (LysM-Cre:GRK2^f^/+ mice) (Willemen et al., 2010).

One issue complicating the exact assessment of the importance of the CX_3_C axis for microglia function in pain perception is that the involved anatomic sites include the spinal cord and DRG that are part of the CNS and peripheral nerve system (PNS) and are hence located behind and in front of the BBB, respectively. Spinal cord and DRG are therefore differentially accessible to the influx of monocytes and contribution of monocyte-derived macrophages (see below).

The emerging recurrent theme of most of the studies addressing the role of the CX_3_C axis in microglia biology is that constitutively expressed membrane-tethered neuronal CX_3_CL1 seems to provide a tonic inhibitory signal to microglia that keeps these cells in a quiescent “sampling” or surveillance mode (Biber et al., 2007) (Figure 1B). Conversely, CX_3_CR1 and CX_3_CL1 deficiencies result in hyper-activated microglia, that depending on the particular setting can be detrimental or beneficial to its environment. As opposed to the homeostatic role of trans-membrane CX_3_CL1, under conditions of challenge such as pain-inducing stimuli, CX_3_CL1 is shed from the neuronal surface and in this context seems to trigger IL-1β production by microglia. Differential activities of shed or membrane-tethered CX_3_CL1 might be related to the recently reported requirement for integrin binding for CX_3_CR1 signaling, although both isoforms interacted with integrins (Fujita et al., 2012). Interestingly, shed, presumably neuronal-derived CX_3_CL1 becomes detectable in serum, when CX_3_CR1 is absent suggesting that CX_3_CR1-expressing cells provide a constitutive sink for it (Cardona et al., 2008). Clearly though, further experimentation will be required to study potential differential effects of membrane-tethered and shed CX_3_CL1 entities on microglial CX_3_CR1 signaling in the *in vivo* context, for instance involving animals manipulated to express exclusively shed CX_3_CL1 (Kim et al., 2011).

## The challenge—microglia vs. monocyte-derived macrophages

The high CX_3_CR1 expression in microglia and resulting bright green fluorescence of CX_3_CR1^gfp^ microglia turned CX_3_CR1^gfp^ mice into a valuable tool to probe microglia function. Moreover, since circulating blood monocytes express CX_3_CR1, these reporter animals became instrumental to solve a long-standing debate about the origins of microglia cells. Microglia are hematopoietic cells that develop independent of neuroectoderm-derived neurons, astrocytes and oligodendrocytes. However, the relationship microglia have with bone marrow-derived macrophages that originate from blood monocytes had long remained a matter of dispute. Irradiation chimeras were generated to define the hematopoietic stem cell (HSC) origin of microglia. Yet, interpretation of the results obtained was confounded by the facts that first microglia are radio-resistant and thus not replaced by a bone marrow graft, and that second the irradiation compromised the BBB allowing monocyte infiltrates. Revision of these studies taking advantage of CX_3_CR1^gfp^ mice to mark either the microglia or the blood compartment and introducing cellular exchange in parabionts and CCR2 dependency as a indication of monocyte-derivation (Mildner et al., 2007; Ajami et al., 2011) have now firmly established that microglia and monocyte-derived brain macrophages are distinct entities. Moreover, these studies were confirmed by fate mapping studies and the demonstration that amoeboid CX_3_CR1/GFP^+^ cells seed the neuroepithelium of the developing brain well before the emergence of “definitive” hematopoietic precursors, at E10.5–E11.5, and following infiltration proliferate and change their morphology into a more branched phenotype (Ginhoux et al., 2010; Swinnen et al., 2013). As opposed to the macrophages colonizing the surface ectoderm these early microglia lack CCR2 expression, established using CX_3_CR1^gfp^:CCR2^rfp^ double reporter mice (Mizutani et al., 2012). Also the spinal cord was shown to be colonized tissue as early as E10.5, although the route by which the microglia precursors reach the respective tissues remain to be resolved (Rigato et al., 2011). The emerging scheme thus holds that microglia are established from primitive macrophages and subsequently maintain themselves through longevity and limited self-renewal independent from further input from definitive hematopoiesis through the HSC-monocyte axis. This prenatally established, hard-wired resident microglia compartment can be complemented on demand by macrophages that arise from monocytes recruited from the blood circulation as a transient “emergency squad” during injury or challenge (Mildner et al., 2009) Depending on the time and route of their arrival to tissues, monocytes can contribute both pro- and anti-inflammatory (Mildner et al., 2009; Shechter et al., 2009), but seem eventually to be purged from the CNS context after resolution of the inflammation (Ajami et al., 2011).

Monocytic infiltrates pose a unique challenge to the study of *bona fide* microglia, as monocyte-derived macrophages become phenotypically indistinguishable from resident microglia, in particular upon activation of the latter. This is particularly evident in the study of neuro-inflammatory disorders, such as the multiple sclerosis model of experimental autoimmune encephalitis (EAE) that involves the BBB breakage and substantial monocyte recruitment (Mildner et al., 2009). Emerging evidence from mixed BM chimeras and parabionts indicates that functional contributions of monocyte-derived cells differ from that of the microglia (Ajami et al., 2011). However, efforts to address these differential functions so far relied largely on the generation of BM chimeras and the manipulation of BM-derived component. Definitive evidence for microglia functions calls for experimental systems that will allow the exclusive manipulation of microglia in non-irradiated mice.

## Genetic manipulation of microglia in context—CX_3_CR1^Cre^ and CX_3_CR1^CreER^ animals

In order to overcome the above constraints of current microglia studies, we decided to exploit the high CX_3_CR1 promoter activity of microglial cells for their genetic manipulation. We generated CX_3_CR1^Cre^ and CX_3_CR1^CreER^ animals (Yona et al., 2013), by targeted insertion of the recombinase genes that mimicked the situation of the CX_3_CR1^gfp^ locus previously shown to tightly reflect endogenous CX_3_CR1 expression (Jung et al., 2000) (Figure 3B). CX_3_CR1^Cre^ mice express constitutively active Cre recombinase resulting in the spontaneous non-reversible rearrangement of loxP site-flanked alleles in CX_3_CR1-expressing cells. In contrast, the CX_3_CR1^CreER^ system comprises a conditional active Cre-ERT2 recombinase that is fused to a mutated ligand-binding domain (LBD) of the human estrogen receptor (ER) (Feil et al., 2009). Two point mutations in the ER-LBD allow binding of the synthetic estrogen antagonist tamoxifen (TAM) and prevents constitutive activation of the CreER by endogenous estradiol (Feil et al., 2009). In the unbound form, the CreER fusion protein resides in the cytoplasm in an inactive complex with heat shock proteins. Upon administration and binding of TAM, the CreER is freed to translocate to the nucleus and mediate the site-specific recombination.

Importantly, CX_3_CR1^Cre^ and CX_3_CR1^CreER^ animals differ considerably with respect to the cells targeted. In CX_3_CR1^CreER^ animals only cells that express CX_3_CR1 and hence the CreER transgene will undergo rearrangement at the time of the TAM treatment. In contrast and as best demonstrated in combination with respective reporter mouse strains (Yona et al., 2013), in CX_3_CR1^Cre^ animals also cells that are derived from CX_3_CR1^+^ cells but subsequently silenced CX_3_CR1 expression will have recombined the loxP-flanked loci. CX_3_CR1^Cre^ animals therefore report on the history of the cell and can be used for fate mapping studies (Yona et al., 2013).

Analysis of CX_3_CR1^Cre^ and CX_3_CR1^CreER^ mice crossed to animals harboring a floxed YFP reporter gene (Srinivas et al., 2001) established that these systems indeed efficiently target CNS microglia (Yona et al., 2013). As expected, in CX_3_CR1^Cre^: YFP mice, and only in TAM-treated CX_3_CR1^CreER^: YFP mice, more than 95% of both brain and spinal cord microglia were found YFP labeled (Figure 3C). Thus, when crossed to mice harboring “floxed” candidate genes, these systems can be utilized to delete or express specific genes in CX_3_CR1^+^ microglia, either straight from their development onwards, using the constitutive Cre system, or in specific time windows, using the inducible Cre system.

Of note, certain lymphocyte subsets and myeloid cells, other than microglia express CX_3_CR1. TAM treatment of CX_3_CR1^CreER^ mice results accordingly also in gene rearrangement in these cells (Yona et al., 2013) (Figure 3D), including monocytes and peripheral macrophages, i.e., exactly the cells that have confounded the functional analysis of the microglia compartment in the previous studies. However, most peripheral myeloid cells that underwent Cre activation and rearrangements have a limited half-life and are hence continuously replaced by BM-derived cells. Genetic modifications, such as the activation of the YFP reporter gene or the deletion of “floxed” alleles are thus progressively lost with time in these populations. In contrast, the resident microglia pool which self-renews without further input from the BM retains once introduced gene modifications throughout the life of the organism (Figure 3D). This feature thus allows generation of animals that harbor specific genetic manipulations restricted to microglia. Of note, there are also other tissue macrophage populations such as Kupffer cells and peritoneal macrophages that are established before birth and then self renew (Schulz et al., 2012; Yona et al., 2013). However, as most of these cells lack CX_3_CR1 expression, they are not targeted by the CX_3_CR1^CreER^ approach in adulthood (Yona et al., 2013). CX_3_CR1^+^ intestinal macrophages on the other hand loose their gene modifications, as they are progressively replaced by monocytes (Zigmond et al., 2012) (Figure 3D). Taken together, the combination of CX_3_CR1^Cre^ and CX_3_CR1^CreER^ animals provides a unique tool to probe for the involvement of microglia in CNS development, CNS maintenance and CNS responses to pathological challenges.

## Conclusion

Microglia are the main representatives of the immune system in the healthy brain as such likely to critically contribute to the brain's resistance to pathological challenges. Moreover, recent data highlight the critical involvement of microglia in CNS development and homeostasis. Given its strategic localization at the neuronal/microglial interface the CX_3_C axis is likely to play a prominent role in these activities. Thus accumulating evidence suggest that neuronal CX_3_CL1 acts as a critical inhibitory signal retaining microglia in quiescent mode and preventing collateral damage due to microglia hyper-activation. Aside from its biological role, the CX_3_CR1 chemokine receptor provides us however also with a unique foothold to study microglia in context using state of the art imaging and gene manipulation approaches. The near future is hence likely to provide valuable insight into contributions of these intriguing cells to brain physiology and might pave the way for the development of microglia manipulation for therapeutic purposes.

### Conflict of interest statement

The authors declare that the research was conducted in the absence of any commercial or financial relationships that could be construed as a potential conflict of interest.
